# A 10-Year Retrospective Study of Inclusion Body Hepatitis in Meat-Type Chickens in Spain (2011–2021)

**DOI:** 10.3390/v13112170

**Published:** 2021-10-28

**Authors:** Kateri Bertran, Angela Blanco, Noelia Antilles, Miquel Nofrarías, Rosa M. Valle, Àlex Cobos, Antonio Ramis, Mar Biarnés, Natàlia Majó

**Affiliations:** 1 IRTA, Centre de Recerca en Sanitat Animal (CReSA, IRTA-UAB), Campus de la Universitat Autònoma de Barcelona (UAB), 08193 Bellaterra, Spain; miquel.nofrarias@irta.cat (M.N.); rosa.valle@irta.cat (R.M.V.); alex.cobos@irta.cat (À.C.); antonio.ramis@uab.cat (A.R.); natalia.majo@irta.cat (N.M.); 2 Centre de Sanitat Avícola de Catalunya i Aragó (CESAC), 43206 Reus, Spain; ablanco@cesac.net (A.B.); nantilles@cesac.net (N.A.); mbiarnes@cesac.net (M.B.); 3 Departament de Sanitat i Anatomia Animals, Facultat de Veterinària, Universitat Autònoma de Barcelona (UAB), Campus de la UAB, 08193 Bellaterra, Spain

**Keywords:** fowl adenovirus, inclusion body hepatitis, Spain, epidemiology, broilers, broiler breeder pullets, phylogenetic analysis

## Abstract

A surge in fowl adenovirus (FAdV) causing inclusion body hepatitis (IBH) outbreaks has occurred in several countries in the last two decades. In Spain, a sharp increase in case numbers in broilers and broiler breeder pullets arose since 2011, which prompted the vaccination of breeders in some regions. Our retrospective study of IBH cases in Spain from 2011 to 2021 revealed that most cases were reported in broilers (92.21%) and were caused by serotypes FAdV-8b and -11, while cases in broiler breeder pullets were caused by serotypes FAdV-2, -11, and -8b. Vertical transmission was the main route of infection, although horizontal transmission likely happened in some broiler cases. Despite the inconsistent and heterogeneous use of vaccines among regions and over time, the number of cases mirrored the use of vaccines in the country. While IBH outbreaks were recorded year-long, significantly more cases occurred during the cooler and rainier months. The geographic distribution suggested a widespread incidence of IBH and revealed the importance of a highly integrated system. Our findings contribute to a better understanding of FAdV infection dynamics under field conditions and reiterate the importance of surveillance, serological monitoring of breeders, and vaccination of breeders against circulating serotypes to protect progenies.

## 1. Introduction

Inclusion body hepatitis (IBH) is an acute disease of chickens usually caused by certain strains of fowl adenovirus (FAdV) species D (FAdV-2, -3, -9, -11) and E (FAdV-6, -7, -8a, -8b) of the genus Aviadenovirus, family Adenoviridae [1]. IBH has a worldwide distribution, affects mainly young broiler chickens up to 5 weeks of age, and is characterized by a sudden increase in mortality, which may occasionally be as high as 30% [1]. Within chickens, broilers are more susceptible to IBH than layers, pointing to the importance of the genetic background [2]. Besides sporadic reports in other domestic birds, including pigeons, quails, ducks, and ostriches [1,3,4], FAdVs have been isolated from wild birds, indicating a possible carryover of the disease into and from wild birds [3,5].

In the last two decades, a growing number of IBH outbreaks due to FAdV-2, -8a, -8b, and -11 have been reported worldwide [1,6,7,8,9,10,11,12,13,14,15]. In Spain, a sharp increase in the number of IBH outbreaks in broiler and broiler breeder pullet flocks has occurred since 2011. We previously performed a molecular and pathobiological characterization of FAdV-8b and -11 isolates from IBH cases in Spain from 2011 to 2013 [16]. A total of 52 IBH cases were diagnosed between 2011 and 2013, with the great majority in broilers (*n* = 45) compared to broiler breeders (*n* = 7). From these, 37 strains were classified as FAdV-8b, while the remaining 15 were classified as FAdV-11 (*n* = 10), -2 (*n* = 4), and -8a (*n* = 1) [16]. Following experimental inoculation of specific pathogen-free layer chickens with FAdV-8b and -11, our results confirmed hepatic lesions and horizontal transmission without overt clinical signs and mortality [16].

The economic losses to the Spanish poultry industry caused by the dramatic increase in IBH cases prompted the use of vaccination in broiler breeders in some regions of the country from the end of 2012 until the first half of 2019. However, the inconsistent use of vaccines across regions and throughout the years has prevented a complete eradication of IBH in Spain, and cases are still being reported. The objective of this retrospective study was to analyze the incidence and distribution of FAdV-causing IBH cases in broilers and broiler breeders in Spain from 2011 to 2021, and to define patterns based on bird type, age, FAdV serotype, time (inter- and intra-annual), and geographic location.

## 2. Materials and Methods

### 2.1. Clinical Cases

A total of 981 clinical cases of suspected IBH were submitted to the Veterinary Pathology Diagnostic Service (College of Veterinary Medicine, UAB), Centre de Recerca en Sanitat Animal (IRTA-CReSA), or Centre de Sanitat Avícola de Catalunya i Aragó (CESAC). All cases were submitted between June 2011 and May 2021 and were collected from commercial broiler and broiler breeder pullet farms in Spain. Cases of suspected IBH were considered when gross hepatic lesions indicative of IBH were observed, i.e., pale, friable, swollen livers with occasionally small white foci and petechial or ecchymotic hemorrhages. Information on submission date, company, farm, location, type of bird, and age were obtained from all suspected cases.

### 2.2. Diagnosis of Suspected IBH Cases

Formalin-fixed, paraffin-embedded (FFPE) liver samples were processed for routine hematoxylin/eosin staining and used for histopathological diagnosis of IBH. Necrotizing hepatitis or hepatocyte degeneration and necrosis with intranuclear inclusion bodies in hepatocytes were indicative of IBH [1].

Mainly frozen liver samples, and occasionally also FFPE liver samples, cloacal swabs, and liver impressions in Flinders Technology Associates (FTA) cards (Whatman Plc., Kent, UK) were used for molecular detection and sequencing of FAdV, as previously described [16]. Briefly, viral DNA was extracted using NucleoSpin Blood kit (Macherey-Nagel, GmbH & Co. KG, Düren, Germany) from frozen livers, cloacal swabs, and FTA impressions, or QIAamp DNA FFPE Tissue kit (Qiagen, Redwood City, CA, USA) from FFPE samples. Viral DNA was detected by PCR targeting 590 base-pairs (bp) of the hexon gene of FAdV [17]. The amplified PCR product was purified using NucleoSpin Gel and PCR Clean-up kit (Macherey-Nagel). Sequencing reactions were performed with BigDye Terminator v3.1 Cycle Sequencing kit (Applied Biosystems, Foster City, CA, USA) and analyzed using 3130xl Genetic Analyzer (Applied Biosystems).

### 2.3. Statistical Analysis

All statistical analyses were performed using GraphPad Prism Version 9. The D’Agostino and Pearson test was used to assess the normality of distribution of investigated parameters. Fisher’s exact test was used for contingency table analyses. Kruskal–Wallis test or Mann–Whitney test was used to analyze significant difference for means. All tests were two-sided and statistical significance was declared at *p* value ≤ 0.05. The *p* values are represented as: * (*p* ≤ 0.05); ** (*p* < 0.01); *** (*p* < 0.001); and **** (*p* < 0.0001).

### 2.4. Phylogenetic Analysis

Phylogenetic analysis of the hexon gene of FAdV field strains and reference strains was performed by ClustalW and maximum-likelihood method with 1000 bootstrap replicates using MEGA 5.2 software. Bioedit 7.2.6 was used to study the nucleotide identities within FAdV strains and obtain the sequence identity matrix.

## 3. Results

### 3.1. Distribution of IBH Cases Based on Poultry Type

**Figure 1:**
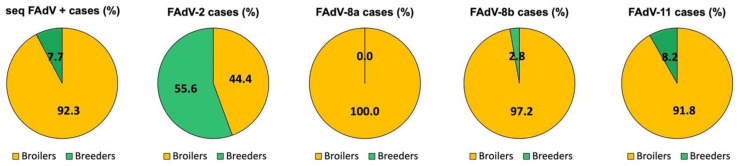
Distribution of sequenced FAdVs and FAdV serotypes by poultry type between 2011 and 2021 in Spain. Percentage of sequenced FAdV positive cases and FAdV serotypes in broilers (yellow) and broiler breeder pullets (green, referred to as Breeders).

Out of the 981 suspected cases submitted between June 2011 and May 2021, 321 cases were confirmed positive for FAdV by PCR and/or histopathology. Among the confirmed cases, 92.2% (296/321) were broiler cases and 7.8% (25/321) were broiler breeder pullet cases. FAdV sequences were obtained from 298 cases out of the 321 confirmed IBH cases, with a similar higher proportion in broilers (92.3%) compared to breeders (7.7%) (Figure 1). Out of all FAdV sequences, the great majority of FAdV-8a, -8b, and -11 were detected in broilers, whereas FAdV-2 was identified at a higher percentage in broiler breeder pullets (55.6%) compared to broilers (44.4%) (Figure 1).

### 3.2. Distribution of IBH Cases in Broilers

**Figure 2:**
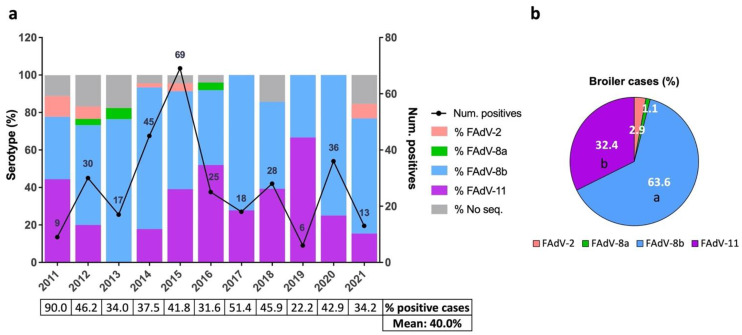
Distribution of IBH cases in broilers between 2011 and 2021 in Spain. (**a**) Number of positive IBH cases in broilers (black line, number of cases per year indicated, (right) *y* axis) and percentage of FAdV serotypes per year (color-coded bars, (left) *y* axis). The percentage of positive cases out of the suspected IBH cases submitted per year and overall is shown as a table below. (**b**) Overall percentage of FAdV serotypes in positive IBH cases in broilers. Different letters designate statistical significance in Fisher’s exact test (*p* ≤ 0.05).

Inter-annual temporal analysis showed an increase in the number of positive broiler cases since June 2011, which peaked in 2015 (*n* = 69) and subsequently decreased, even though spikes were identified in 2018 (*n* = 28) and 2020 (*n* = 36) (Figure 2a). The mean of positivity rates of broiler suspected IBH cases was 40%, ranging from 22.2% (2019) to 90% (2011) (Figure 2a). The distribution of serotypes was similar over the years; FAdV-8b and -11 were consistently identified as the main circulating serotypes, while FAdV-2 and -8a were sporadically detected (Figure 2a). Overall, FAdV-8b was the most frequent serotype detected (63.6%), followed by FAdV-11 (32.4%), FAdV-2 (2.9%), and FAdV-8a (1.1%) (Figure 2b).

### 3.3. Distribution of IBH Cases in Broiler Breeder Pullets

**Figure 3:**
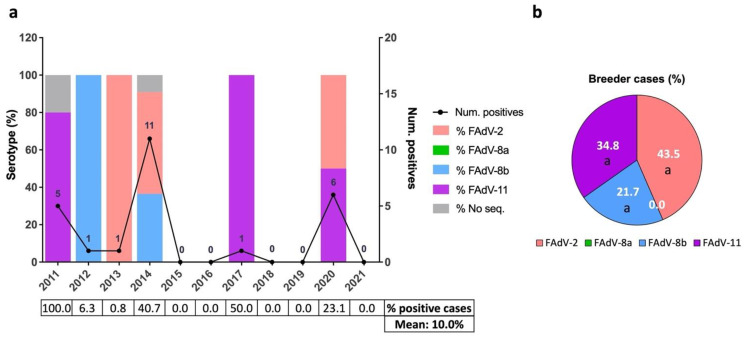
Distribution of IBH cases in broiler breeder pullets between 2011 and 2021 in Spain. (**a**) Number of positive IBH cases in broiler breeder pullets (black line, number of cases per year indicated, (right) *y* axis) and percentage of FAdV serotypes per year (color-coded bars, (left) *y* axis). The percentage of positive cases out of the suspected IBH cases submitted per year and overall is shown as a table below. (**b**) Overall percentage of FAdV serotypes in positive IBH cases in broilers. Different letters designate statistical significance in Fisher’s exact test (*p* ≤ 0.05).

Positive broiler breeder pullet cases were sparsely detected inter-annually, with 2011 (*n* = 5), 2014 (*n* = 11), and 2020 (*n* = 6) the years when most cases were diagnosed (Figure 3a). The positivity rates of broiler breeder pullet suspected IBH cases were very erratic due to the low number of cases (Figure 3a). The distribution of serotypes was equally inconsistent over the years (Figure 3a). Overall, FAdV-2, -11, and -8b were detected in similar percentages (Figure 3b). Serotype FAdV-8a was not detected in broiler breeder pullets.

### 3.4. Intra-Annual Seasonality of IBH Cases

**Figure 4:**
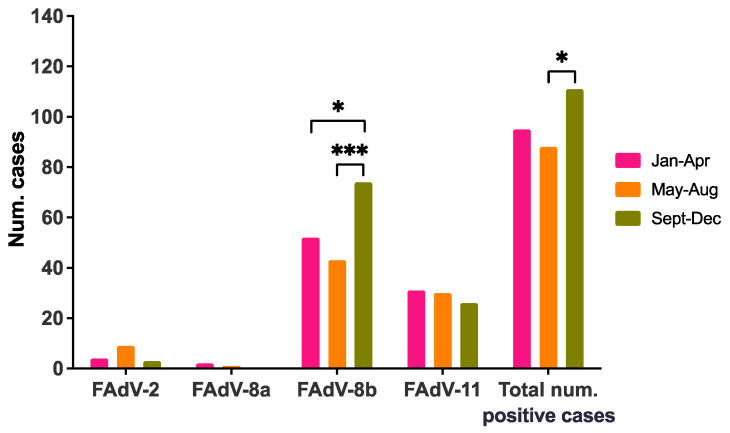
Intra-annual seasonality of IBH cases in meat-type chickens in Spain. Distribution of FAdV serotypes and number of positive IBH cases per quadrimester. Only whole years were considered (i.e., 2011 and 2021 were not included). Statistical significance was declared at *p* ≤ 0.05. The *p* values are represented as: * (*p* ≤ 0.05); and *** (*p* < 0.001).

IBH cases were classified into three quadrimesters based on submission date. Only whole years were considered (i.e., 2011 and 2021 were not included). Intra-annual distribution showed a higher number of IBH cases in the third quadrimester (September–December) compared to the rest of the year, which was significantly higher only when compared to May–August (Figure 4). Similarly, a significant increase in FAdV-8b cases were detected at the end of the year compared to the other two earlier quadrimesters; such a trend was not observed for any other serotype (Figure 4).

### 3.5. Geographic Distribution of IBH Cases

**Figure 5:**
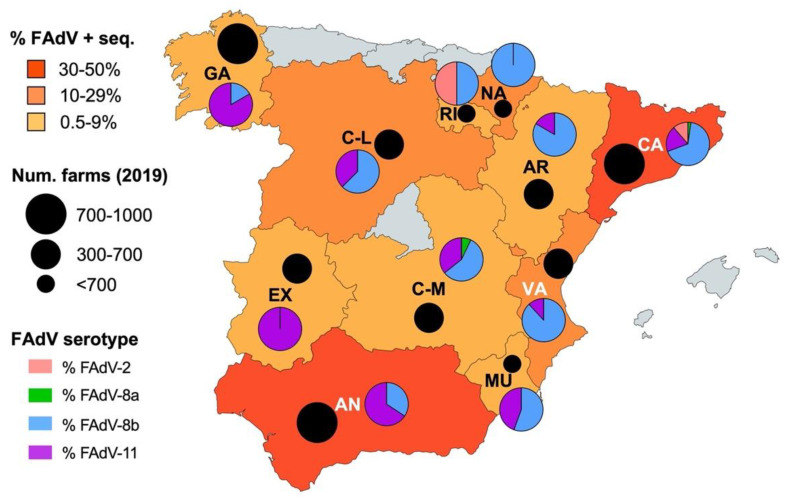
Geographic distribution of IBH cases and FAdV serotypes in meat-type chickens between 2011 and 2021 in Spain. The colors correspond to the percentage of FAdV-positive sequenced samples from each region. The pie charts indicate the percentage of FAdV serotypes in each region. The black circles indicate the number of meat-type poultry farms in each region according to the 2019 census [18].

IBH cases for which FAdV serotype was available were classified based on region. IBH cases were submitted from most regions of Spain, with Catalonia (46.1%) and Andalusia (36.6%) the regions with the highest prevalence of positive sequenced IBH cases (Figure 5). Interestingly, a geographical distribution based on serotype was observed; FAdV-8b serotype tended to circulate in North-Eastern regions, while FAdV-11 was more prevalent in South-Western regions (Figure 5).

### 3.6. Distribution of IBH Cases Based on Age

Infections occurring before the waning of maternal antibodies (i.e., at around 18–24 days of age) are likely caused by vertical transmission, while infections at a later age normally involve horizontal transmission [1,3,6]. In order to infer the contribution of vertical versus horizontal transmission, IBH cases were classified based on age at time of diagnosis. Most broiler cases affected birds <21 days of age (53.8%), which represented a significantly higher percentage than cases in either of the other two age brackets, i.e., 21–30 days and >30 days. When cases were distributed between only two age brackets (<21 days vs. >21 days), no statistical difference between age groups was observed. In contrast, a significantly higher proportion of broiler breeder pullet cases were detected in birds <21 days of age (76.2%) compared to cases at 21–30 days (23.8%). Cases at >30 days were not detected in any broiler breeder pullet. When comparing the mean age at time of diagnosis, broilers were affected at a significantly older age (20.9 days) than broiler breeder pullets (14.6 days) (*p* < 0.0001).

**Figure 6:**
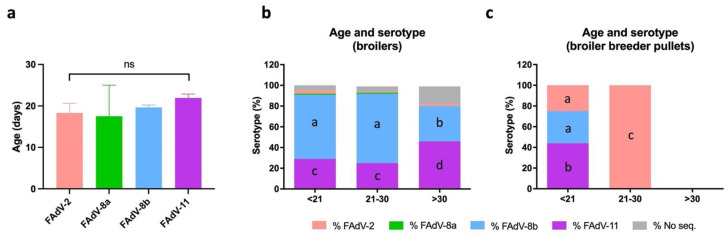
Distribution of FAdV serotypes based on age in meat-type chickens between 2011 and 2021 in Spain. (**a**) Mean age of IBH-positive broilers and broiler breeder pullets based on FAdV serotype. Statistical significance was declared at *p* ≤ 0.05. (**b**) Percentage of FAdV serotypes per age group in broilers. (**c**) Percentage of FAdV serotypes per age group in broiler breeder pullets. Different letters designate statistical significance in Fisher’s exact test (*p* ≤ 0.05).

Although no statistical differences were observed among FAdV serotypes based on age (Figure 6a), different serotype distribution among age groups was observed when bird type was considered (Figure 6b,c). In broilers, FAdV-8b was the most prevalent serotype in birds <21 days (62%) and at 21–30 days (67%), while FAdV-11 cases were significantly more prevalent at >30 days (46%) (Figure 6b). In contrast, FAdV-11 was the most prevalent serotype in broiler breeder pullets <21 days (44%), despite serotypes 2 and 8b being 25% and 31%, respectively (Figure 6c). Interestingly, FAdV-2 was the only serotype detected at 21–30 days (Figure 6c).

### 3.7. Phylogenetic Analysis

Out of the 298 FAdV sequences obtained throughout the 10-year period, 246 clean hexon gene region sequences were used for the phylogenetic analysis (final alignment length of 470 bp) (Figure S1). The FAdV strains detected were genetically homogeneous within each genotype and to the corresponding reference strain in the considered hexon gene region. Nucleotide identities between FAdV-2 field strains and the reference strain 685 (AF508947) were 97.1–96.9%, and identities within the FAdV-2 field strain cluster ranged from 100–98.4%. The maximum nucleotide identities observed between FAdV-8a field strains and the reference strain TR59 (EU979374) were 98.4–97.4%, and identities within the FAdV-8a field strain cluster were 98.4%. Nucleotide identities of 99.7–94.8% were observed between FAdV-8b field strains and the reference strain 764 (EU979375), with a 100–94.8% nucleotide identity within the FAdV-8b field strain cluster. Maximum nucleotide identities of 97.3% were detected between FAdV-11 field strains and the reference strain UF71 (EU979378), and no differences (i.e., 100% of nucleotide identity) were observed within the FAdV-11 field strain cluster.

## 4. Discussion

Since 2011, Spain experienced an increase in the number of IBH outbreaks in meat-type commercial birds [16], with no exception to the worldwide rising trend of the last two decades [1,6]. Despite vaccination campaigns, IBH cases continue to be reported in Spain. In this 10-year retrospective study, we aimed to analyze the incidence and distribution of FAdV-causing IBH cases in broilers and broiler breeders in Spain considering bird type, age, FAdV serotype, and temporal and geographical patterns. It is worth highlighting that the number of diagnosed IBH cases in the present study cannot be representative of the actual prevalence of the disease in the country.

The higher incidence of FAdV over the last decades has been speculated to be linked to the efficacy of the elevated standards of biosecurity in preventing FAdV natural infection in breeder stocks [3,6,7]. Starting in 2010, biosecurity in breeder holdings increased substantially in Spain and other European countries due to Salmonella control programs [19,20]. The isolation of breeder flocks in the pullet phase can prevent natural FAdV infection, which can cause two cascade effects: (1) lack of immunity in parental birds and therefore absence of maternally-derived immunity in the progeny; and (2) higher probability of a FAdV infection during the production period, followed by vertical transmission of the virus until sufficient maternal antibodies are developed to prevent vertical transmission [3,6,7]. Here, the great majority of FAdV-causing IBH cases were detected in broilers, suggesting a twofold effect derived from stricter biosecurity standards in breeder farms and lack of the same biosecurity standards in broiler farms. Interestingly, several factors suggest that FAdVs seem to be primary pathogens in the great majority of IBH cases in Spain: (1) the opportunistic chicken anemia virus (CAV) and infectious bursal disease virus are successfully controlled in commercial meat-type poultry by vaccinating broiler breeders; and (2) in the first years, IBH-suspected cases were also screened for CAV, with consistently negative results. These observations build upon existing evidence that FAdV can be primary pathogens of IBH [3].

Over the past 10 years, three different inactivated vaccines were given a national license in Spain to be administered in broiler breeder pullet farms: (1) a commercial vaccine with FAdV-4 and -5 serotypes (ibh 4/8, Avimex, Mexico), administered from the end of 2012 to the end of 2013; (2) an autogenous commercial vaccine derived from 2011–2013 circulating FAdV-8b and -11 serotypes (Vaxxinova, The Netherlands), administered from 2014 to the first semester of 2019; and (3) a commercial vaccine with FAdV-4, -8b, and -11 serotypes (Oleovac HCI Plus+, Farvet, Peru), recently licensed in February 2021. The aim of vaccination of breeder flocks in the pullet phase is primarily to prevent vertical transmission and to protect day-old chicks by maternal antibodies [1,3]. Although the use of vaccines was inconsistent and heterogeneous among Spanish regions and throughout the years (e.g., some regions never adopted the measure, other regions adopted vaccination later or for a shorter time), the evolution of the number of cases seems to mirror the use of FAdV vaccines in the country, with the number of cases peaking approximately three times, i.e., 2011–2012, 2014–2015, and 2020, over the 10-year period in both types of birds. The FAdV types detected during this time belonged to species D (FAdV-2 and -11) and E (FAdV-8a and -8b), mirroring the serotypes mostly associated with IBH globally [3,6,21]. Regardless of age, broilers were mostly affected by FAdV-8b and -11, while broiler breeder pullets had a similar prevalence of FAdV-2, -8b, and -11, akin to previous observations [22,23].

The partial effectiveness of the inactivated vaccine used in 2012–2013 would indicate some heterologous protection provided by a different FAdV species (FAdV-4, species C) or a different serotype within the same species (FAdV-5, species D) compared to the strains circulating in broiler breeder pullets at that time (i.e., FAdV-2 [species D] and -8b [species E]). The heterogeneity of IBH-causing FAdV types in Spain, being FAdV-D and -E species, argues for the use of vaccines combining serotypes from these two species, which has been shown to elicit adequate progeny protection against various challenge strains [3,24,25]. Although heterologous protection in FAdV-causing IBH is still under debate [1,6], previous experimental studies suggest that FAdV-4 inactivated vaccines could provide broad cross-protection against various serotypes of FAdV, not only in vaccinated breeders but also in their progenies [3,26], in line with our findings. However, the cross-protective efficacy of FAdV-4 vaccines in the field has yielded conflicting results; while a field study in China suggests that FAdV-4 inactivated vaccine successfully controlled FAdV-11 [27], its application in South Korea caused an increase of FAdV-11 cases [28], and no cross-protection against FAdV-8b was observed in either country [27,28]. Thus, the complete absence of FAdV-11 in breeders in 2012–2013 provides a strong field evidence for cross-protection by the inactivated vaccine containing FAdV-4. In view of the sharp decline in the number of cases from 2014 to 2019, it appears that the autogenous vaccine with FAdV-8b (species E) and -11 (species D) was effective in controlling IBH in the country. This was probably due to the high similarity between the vaccine and the circulating strains when using autogenous vaccines, which calls for the need for characterizing prevalent FAdV serotypes when designing successful vaccine programs [21]. Unfortunately, the effects of the last vaccine licensed in Spain are not fully evident yet. Nonetheless, promising results are expected with the use of this trivalent-serotype vaccine combining the circulating FAdV-8b (species E) and FAdV-11 (species D) in addition to the cross-protective FAdV-4 (species C).

Although IBH outbreaks were recorded year-long, the number of IBH cases were significantly higher between September and December. Despite Spain having great variation in climate conditions across regions, these months tend to combine low temperatures with high rainfall [29,30]. Previously, an increased number of IBH outbreaks in broilers were recorded during the cooler and wetter months in India [31] and Pakistan [32], and concomitant problems related to these environmental conditions, such as mycotoxins in feed, have been speculated to be indicators of IBH [31]. We observed that the higher number of cases in the third quadrimester was specifically associated with FAdV-8b cases, a serotype mostly found in broiler farms, which typically implement lower biosecurity standards compared to broiler breeder farms. Although modern, intensive broiler holdings can very accurately control temperature and humidity regardless of the weather conditions, other holdings with not so up-to-date environmental monitoring systems may be unsuccessful in doing so, both in the poultry house and in the feed storage facility. Further studies linking the presence of FAdV and mycotoxins in feed or other problems related to environmental conditions are needed to confirm this hypothesis.

Bearing in mind the limitations of this retrospective study using diagnostic samples, the observed geographic distribution of FAdV-causing IBH cases indicates that the disease is present in virtually all poultry meat-producing regions in Spain in proportionate numbers according to their farm census [18], suggesting a widespread incidence of IBH within the Spanish poultry sector. This finding also reveals the impact of sourcing broilers from a few broiler breeder producers, which happens with the Spanish breeder and broiler industries that are mostly linked in integrated production. Interestingly, a specific geographic distribution of certain serotypes was found, mainly FAdV-8b in North-Eastern regions and FAdV-11 in South-Western regions. Although the reasons behind this geographic distribution of serotypes remain unclear, the epidemiological scenario of FAdVs in neighboring countries could be of importance [7]. The identification of FAdV-11 in Morocco (south of Spain) [10] could indicate circulation of FAdVs via commercial poultry connections, either from Spain to Morocco or vice versa. The lack of publicly available reports of FAdV circulating strains from Portugal (west of Spain) and France (northeast of Spain) prevents confirmation of this type of epidemiological link.

Differences in IBH and FAdV serotype prevalence emerged when age was considered, exposing the different contributions of vertical vs. horizontal transmission. Both transmission routes are involved in FAdV epidemiology and, although no clear differentiation between them can always be made, a clinical sign onset within 21 days of age is more compatible with vertical rather than horizontal infection [1,7]. In the absence of a direct link between infected broilers and their breeder source due to confidentiality from integration companies, both vertical and horizontal transmission of the virus are possible [23]. However, the majority of IBH outbreaks occurred in broilers and broiler breeder pullets younger than 21 days of age, suggesting that vertical transmission was the main route of infection in both types of bird. Interestingly, horizontal transmission was also involved in some broiler IBH cases, again exposing epidemiological patterns derived from different biosecurity standards. Previous studies have reported similar FAdV-causing IBH outbreaks in broilers via a combination of both vertical and horizontal routes [7,23,33]. Importantly, FAdV-causing IBH was not reported in mature birds, in line with the literature [1], although the virus can be reactivated during egg production of breeders and cause subclinical infection while ensuring vertical transmission to the progeny [1].

Our phylogenetic analysis suggested very high uniformity of FAdV strains within each genotype throughout the 10-year period. This finding demonstrates lack of temporal or geographic patterns in the face of viral genetic changes and supports the existence of strong epidemiological links between integrated farms in the country. Further studies examining larger regions of the hexon gene are needed to infer viral evolution in more detail.

Different age-related prevalence of FAdV serotypes were observed. On the one hand, FAdV-8b was the most prevalent serotype in younger broilers, with FAdV-11 cases increasing in birds >30 days. On the other hand, FAdV-11, -8b, and -2 affected younger broiler breeder pullets, but FAdV-2 was the only serotype in older birds. These age–serotype patterns could be a proxy of the epidemiological picture of FAdV in the poultry meat-type industry in Spain. Presumably, in years with no or little vaccination or in those regions not adopting the measure, FAdV-2, -8b, and -11 could be circulating in broiler breeder pullet holdings following vertical introduction, with FAdV-2 becoming predominant in older pullets thanks to horizontal spread. Consequently, antibodies against this serotype would be elicited in most of these broiler breeder pullets. Maternal antibodies mainly against FAdV-2 would be passed to their progeny as broiler breeder pullets enter production, but also FAdV-8b and -11 could be transmitted vertically to broilers. Interestingly, FAdV-2 is virtually absent from broiler flocks, possibly thanks to the maternal immunity against this serotype. Maternal antibodies against FAdV-2 could also be responsible for the higher prevalence of FAdV-8b in younger broilers compared to FAdV-11. This would indicate better cross-protection by FAdV-2 (species D) antibodies against FAdV-11 (also species D) compared to limited cross-protection against FAdV-8b (species E). Finally, as maternal immunity against FAdV-2 may decline in older broilers, the prevalence of FAdV-11 would increase. Although our observations suggest that this is a plausible scenario of the IBH epidemiology in the country, in certain regions, and/or for most years, it is relevant to bear in mind the limitations of combining 10 years of data at the expense of losing the detail that annual tendencies can provide. Likewise, difficult traceability from grandparent stocks prevents us from drawing more well-supported conclusions, particularly concerning the vertical transmission of FAdV viruses to broiler breeder pullets.

## 5. Conclusions

In this 10-year retrospective study we analyzed the incidence and distribution of FAdV-causing IBH cases in meat-type commercial poultry in Spain. Our findings contribute to better understand FAdV infection dynamics under field conditions, the impact of vaccination, and the ramifications of an integrated breeder and broiler production of a specific geographic region. Our observations reiterate the importance of biosecurity, surveillance, serological monitoring of breeders, and vaccination against circulating serotypes for maternal antibody production in breeders to protect progenies.

## Supplementary Materials

The following are available online at https://www.mdpi.com/article/10.3390/v13112170/s1. Figure S1: Maximum-likelihood phylogenetic tree.
