# Supplementary material for: A 10-Year Retrospective Study of Inclusion Body Hepatitis in Meat-Type Chickens in Spain (2011–2021)
Source: Viruses. 2021 Oct 28;13(11):2170. doi: 10.3390/v13112170 (PMC8617850; doi:10.3390/v13112170)
Supplement: Supplementary file 1 [file viruses-13-02170-s001.zip › viruses-1434093-supplementary.pdf]

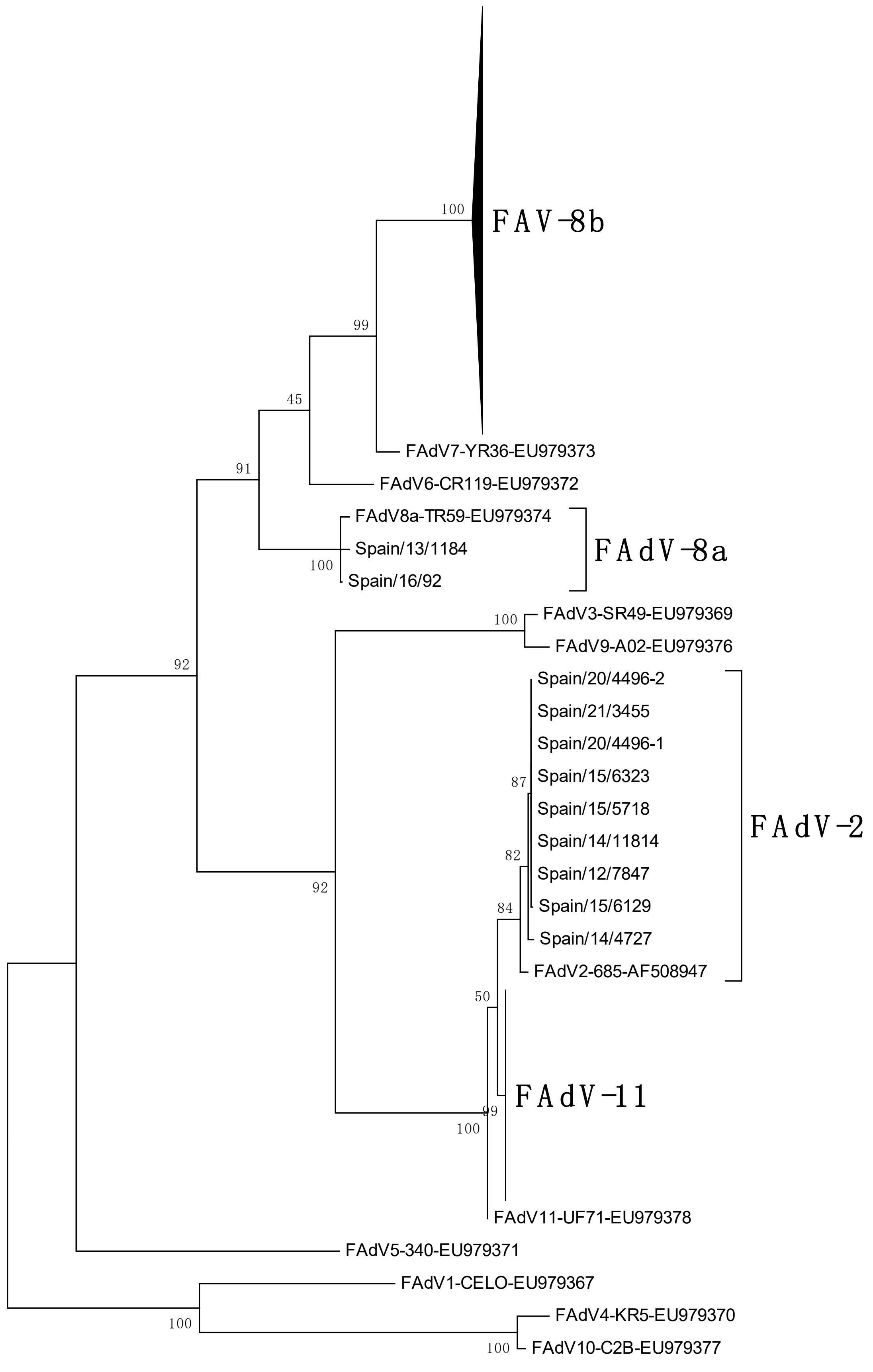

100
FAV-8b
99
45
FAdV7-YR36-EU979373
91
FAdV6-CR119-EU979372
FAdV8a-TR59-EU979374
Spain/13/1184
100
Spain/16/92
FAdV-8a
100
FAdV3-SR49-EU979369
FAdV9-A02-EU979376
92
Spain/20/4496-2
Spain/21/3455
Spain/20/4496-1
Spain/15/6323
87
Spain/15/5718
Spain/14/11814
FAdV-2
82
Spain/12/7847
92
Spain/15/6129
84
Spain/14/4727
FAdV2-685-AF508947
50
FAdV-11
99
100
FAdV11-UF71-EU979378
FAdV5-340-EU979371
FAdV1-CELO-EU979367
100
FAdV4-KR5-EU979370
100
FAdV10-C2B-EU979377
0,1

Figure S1. Maximum-likelihood phylogenetic tree. Maximum-likelihood phylogenetic tree with 1000 bootstrap replicates showing the relationships among the nucleotide partial sequences of the FAdV hexon gene (final alignment length 470 bp). FAdV strains (n=246) detected from June 2011 to May 2021 from commercial meat-type chickens in Spain and main reference FAdV serotype strains (n=12) are compared. Horizontal branches indicate the sequence distance (number of nucleotide substitutions per site) and are drawn to scale.
